# Laser Micromachining for the Nucleation Control of Nickel Microtextures for IR Emission

**DOI:** 10.3390/mi16060696

**Published:** 2025-06-11

**Authors:** Tatsuhiko Aizawa, Hiroki Nakata, Takeshi Nasu

**Affiliations:** 1 Surface Engineering Design Laboratory, Shibaura Institute of Technology, Tokyo 144-0056, Japan; 2 EBINAX Co., Ltd., Tokyo 144-0033, Japan; nakata-h@ebinadk.com (H.N.); nasu-t@ebinadk.com (T.N.)

**Keywords:** femtosecond laser microtexturing, nucleation site control, wet nickel microtexturing, micro-pillar texture alignment, infrared emission, heat radiation

## Abstract

Femtosecond laser micromachining was utilized to build up a micro-through-hole array into a sacrificial film, which was coated onto a copper specimen. This micro-through hole was shaped in the paraboloidal profile, with its micro-dimple on the interface between the copper substrate and the film. This profile was simply in correspondence with the laser energy profile. The array was used as a nucleation and growth site for nickel cluster deposition during wet plating. The micro-pillared unit cells nucleated at the micro-dimple and grew on the inside of the micro-through hole. After removing the sacrificial film, cleansing, and polishing, the nickel micro-pillar array was obtained, standing on the copper substrate. These unit cells and their alignments were measured through scanning electron microscopy and laser microscopy. Thermographic microscopy with FT-IR was utilized to measure the IR emittance as a function of wavelength. The focused areas were varied by controlling the aperture to analyze the effects of arrayed microtextures on the IR emittance.

## 1. Introduction

Infrared optics has developed into a new technological keyword, not only in terahertz telecommunication [1] and thermographic imaging [2], but also in heat radiation transfer [3]. Recently, new IR optical devices, which emit electromagnetic waves in the IR range with wavelength selectivity, have been reported in the literature. Nanophotonic control [4,5] and metamaterial design [6,7] exploit the selectively patterned nanostructure of photovoltaic materials. Regularly aligned microcavity texturing [8] and semi-regularly aligned micro-acicular texturing [9,10,11] have heat radiation capacities with equivalent wavelengths to the microcavity depth and micro-cone height, respectively. This success in emitting IR waves from a microtextured device proves that micro- and nano-textured aluminum and copper sheets work as resonators to emit electromagnetic waves with selective wavelengths from the heat source.

Using the acicular micro-/nano-texturing approach, micro-cone unit cells are semiregularly shaped so that their height (H), root diameter (B), and mutual pitch (D) were statistically distributed on each textured copper sheet. Although the resonating wavelength λ (=λ_average_ ± Δλ) had a good correlation with H (=H_avearge_ ± ΔH) by λ_average_ = 2 × H_avearge_ and Δλ = 2 × Δλ [12], the intensity in emissivity, I(λ), was lower than that reported in the literature [4,5,6], e.g., I(λ)~95% for 1 μm < λ < 5 μm in [4,5,6], but I(λ)~30% for 1 μm < λ < 8 μm in [11]. In order to increase this emissivity, I(λ), a unit cell was regularly aligned on the substrate so that IR was efficiently emitted on the regular microtextures, just like a meta-material with low contribution of clearance among unit cells to IR emission. In the present paper, a femtosecond laser machining system is utilized to build up the through-hole array into a sacrificial film, coated on the copper substrate. With well-defined control of the laser beam profile, the copper in the vicinity of the interface to the sacrificial film is also removed using an ablation process to form a micro-dimple at the bottom of the through hole. This micro-dimple is utilized as a nucleation and growth site of nickel textures during wet plating. In general, this wet-plating process is controlled by the total current and current density. The deposition and growth rates of micro-pillars are mainly determined by these two parameters.

**Figure 1:**
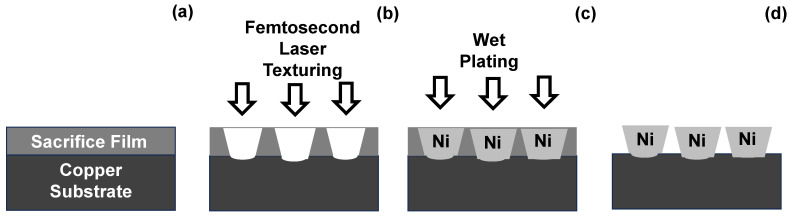
A production line from the sacrificial film, coated on the copper specimen, to the micro-cone textured device. (**a**) Preparation of the sacrificial film on the copper specimen, (**b**) formation of a micro-through-hole array into the specimen, (**c**) formation of nickel textures onto the copper substrate, and (**d**) removal of sacrificial film before polishing and cleansing.

Figure 1 illustrates this procedure to form the nickel microtexture array on the copper substrate via the laser machining and wet-plating processes. The unit cell height in the microtextures is limited by the sacrificial film thickness, as shown in Figure 1a. Otherwise, the unit cells are linked like a flange by overfilling the through-hole array during wet plating. The unit cell size is predetermined by the laser beam profile control, as shown in Figure 1b, e.g., the micro-dimple diameters are tailored to be 2.5 μm to 5 μm and machined into the copper sheet at the bottom of the through-hole array. In most cases of wet-plating processes, each unit cell’s geometry is formed to fill the through-hole cavity. Each sub-cell nucleates on the three-dimensionally growing unit cell surface and coalesces with the others to deposit a new layer. As illustrated in Figure 1c, a unit cell is shaped by filling a through-hole cavity. As noted in [10], this microtexture unit cell geometry is controlled by the mass balance between the deposition rate and the local solution content. With a decreasing local deposition rate, the through-hole is partially filled by nickel clusters to modify the unit cell geometry to a nickel micro-cylinder, a nickel micro-truncated-cone, or a nickel cone. Further electro-chemical solution design and pulse–current–density control in wet plating is needed to modify this unit cell geometry.

The fabricated nickel-textured copper specimen was analyzed via SEM (scanning electron microscopy), as well as LM (laser microscopy). The through-hole of the sacrificial film and the nickel unit cell were measured using these methods. Thermographic microscopy with FT-IR (Fourier Transform Infrared spectroscopy) was employed to measure the IR emission spectrum for the microtextured copper plate. The bare copper plate was also used as a reference to measure this IR emissivity intensity as a function of wavelength. This high IR spectrum characterizes the superiority of the microtextured surface to a bare metallic plate.

## 2. Materials and Methods

An oxygen-free copper plate with a purity of 99.98% and a thickness of 0.5 mm was prepared, cut into square specimens, and mechanically polished before the polyethylene film was spin-coated. This film, with a thickness of 15 μm, was used as a sacrificial film. The femtosecond laser machining system was employed to build up the micro-through-hole array into the specimen. The electro-chemical process was used to nucleate and feed the nickel micro-pillared textures selectively into this array. Both the array subjected to laser microtexturing and the micro-pillar alignment subjected to wet microtexturing were characterized in geometry and dimension. Thermographic microscopy with FT-IR was utilized to measure the IR emissivity of a nickel micro-pillar textured specimen.

### 2.1. Femtosecond Laser Microtexturing Method

**Figure 2:**
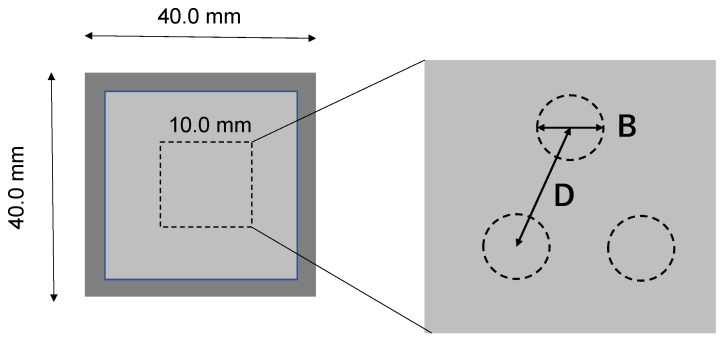
Alignment design on the original microtextures to be laser-machined.

A femtosecond laser machining system (Lipsworks, Co., Ltd., Tokyo, Japan) was utilized to make a through-hole array into a sacrificial film, coated on a square copper specimen with a size of 40 mm and a thickness of 0.5 mm. The wavelength was 515 nm, the average power in maximum was 8.2 W, the pulse duration was 185 fs, the repetition rate was 600 kHz, and the scanning speed was 2000 mm/s. The spot size of a laser beam in focusing was estimated to be 14 μm using a Galvano-meter. The number of pulses (N) was controlled to laser-drill each through-hole. As illustrated in Figure 2, two types of through-hole arrays were designed and transformed from their CAD (Computer-Aided Design) data to CAM (Computer-Aided Machining) data for positioning control in laser machining operations. One is a through-hole array design with a unit hole diameter (B) of 5 μm and a pitch (D) of 3 × B between adjacent through-holes in the triangular lattices. The other is also a through-hole array design with B~2 μm and D~3 × B. In the following, each sample is denoted as A-specimen and B-specimen, respectively. N = 50 on average for the A-specimen, while N = 100 on average for the B-specimen as well.

The laser power and pulse conditions were adjusted to penetrate the sacrificial film thickness and form a surface dimple at the interface between the sacrificial film and the copper substrate. To be discussed later, the through-hole can be shaped to have a paraboloidal side surface with the bottom diameter by B but the top diameter of B’ (>B) since the laser pulse has a Gaussian energy profile.

### 2.2. Nickel Wet Plating Method

Wet plating was employed to selectively deposit the nickel into this through-hole array. NiCl_2_ solution was used as a source of nickel deposits. The total current, the current density, and the chemical solutions influence the nucleation and growth processes to form this nickel unit cell. In the following experiments, they were controlled to drive two-dimensional growth in the through-hole array; the current density was 2.5 A/dm^2^, the plating area was 10 mm × 25 mm (=0.025 dm^2^), the duration was 1.2 ks, and the pH of the solution was controlled to be 2 to 4. The temperature of the solution was held constant at 338 K under air stirring.

### 2.3. Geometric Characterization of Microtextures

SEM (JEOL, Tokyo, Japan) and LM (Keyence, Tokyo, Japan) were utilized to describe the surface profile of the laser-machined through-hole array as well as its cross-sectional profile. They were also used to analyze the surface and cross-sectional profile of the wet-plated unit cell array.

### 2.4. IR Emissivity Measurement

**Figure 3:**
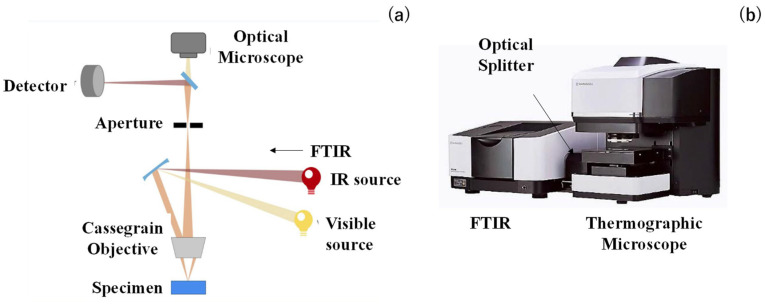
IR emissivity measurement system using thermographic microscopy with FT-IR. (**a**) Optical beam line setting for measurement and (**b**) overview of the entire system.

Thermographic microscopy with FT-IR (Shimazu, Co., Ltd., Kyoto, Japan) was utilized for the measurement of IR emissivity. The optical beam line in this system is explained in Figure 3a. Two beams from IR and visible sources were introduced into the Cassegrain objective, which consists of concave primary and convex secondary mirrors. The beam from this objective was selected by an aperture and detected using a T2SL (Type II Super-Lattice) sensor, as well as the optical microscope. In the following, the aperture was fixed to be 200 μm, although it was controllable to focus the measured area on the microtextured specimen. In practical operation, the thermographic microscope with FT-IR shown in Figure 3b was utilized to measure the IR emittance spectra for the regularly aligned microtextures as well as the bare copper sheet.

## 3. Results

### 3.1. Femtosecond Laser Microtexturing

An A-specimen was prepared for laser microscopy to measure the laser textures on the sacrificial film surface and the laser-machined trace on the interface between the sacrificial film and the copper substrate at a depth of 15 μm.

**Figure 4:**
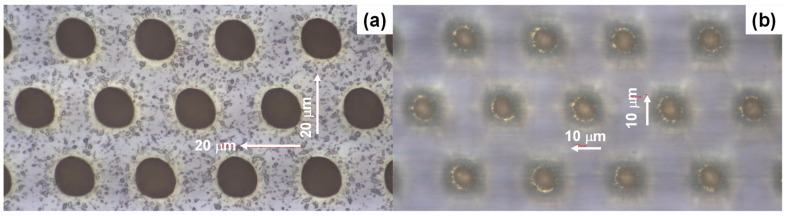
Laser microscopic images of the laser microtextured sacrificial film, which was coated on the copper specimen. (**a**) LM image focused on the film surface and (**b**) LM image focused at a depth of 15 μm from the film surface.

**Figure 5:**
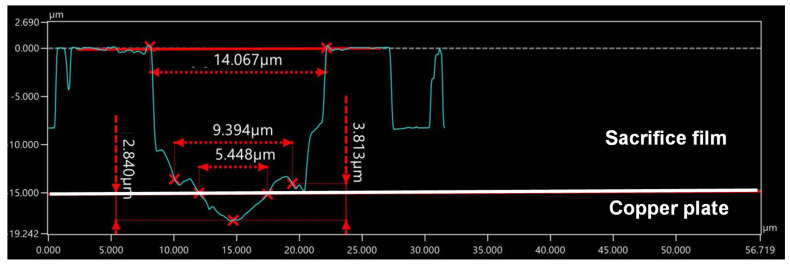
Laser microscopic image of the cross-section of the micro-through-hole array to measure each unit texture profile and the micro-dimple formed into the copper substrate.

As shown in Figure 4a, the top diameter of the through-hole reaches 14 μm, or, B’ = 14 μm, while the bottom diameter reaches around 5 μm, or B = 5 μm in Figure 4b. This difference in the through-hole diameter comes from the laser energy profile. As studied in [12], the femtosecond laser pulse has a Gaussian energy profile so that the through-hole side surface is shaped to be paraboloidal, whereby the diameter of the hole at the inlet becomes larger than that at the top, or, B’ > B. Let us prove this estimate by observing the cross-sectional profile of the through-hole. As depicted in Figure 5, the through-hole side surface is also shaped in a paraboloidal manner corresponding to this energy profile. A micro-dimple with an outer diameter of 5 μm was formed into the copper plate. This micro-dimple was utilized as a nucleation and growth site of nickel micro-pillar unit cells during wet plating after cleansing and ultrasonic polishing. The pitch (D) was estimated from Figure 4 to be 45 μm; D is just given by D~3 × B’.

In a similar manner, the B-specimen was prepared to have a through-hole array with average top and bottom diameters of 13 μm and 3.5 μm, respectively, or, B’ = 13 μm and B = 3.5 μm. To be discussed later, the minimum diameter of the micro-dimples was determined using the focused energy beam profile in femtosecond laser machining.

### 3.2. Nickel Microtexturing via Wet Plating

Nickel wet plating was employed to feed the nickel clusters into the through-holes in the sacrificial film and to fill them. Both the total current and the current density were specified to control the nucleation and growth processes of nickel clusters on the inside of the through-holes. After wet plating, the film was chemically removed to leave the nickel microtextures on the copper sheet. In this chemical removal process, the specimen was dipped into acetone for 300 s under ultrasonic polishing with a frequency of 42.5 kHz.

**Figure 6:**
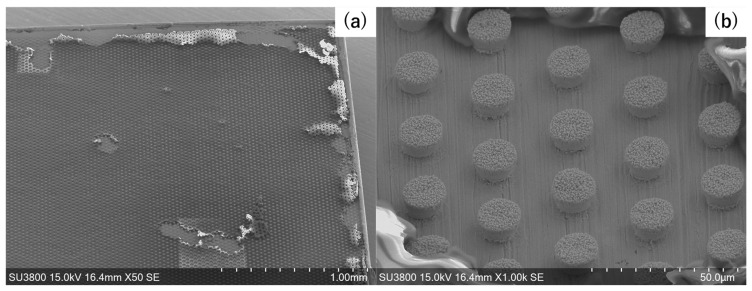
SEM image of the nickel microtexture array after partially removing and polishing the sacrificial film. (**a**) A copper sheet specimen with a nickel micro-pillar array and (**b**) nickel pillar alignment.

Figure 6a depicts the surface condition of the microtextured copper sheet after partial removal of the sacrificial film. The microtexture alignment is formed simply in the same position as through-holes without unfilled holes using wet plating. In the high-magnification SEM image, as shown in Figure 6b, the nickel pillars align themselves in the triangular lattice structure. Their top diameter (B”) becomes B” = 14 μm; B” = B’. The nickel clusters fully fill the through-hole. The pitch (D”) reaches 45 μm; D” = D.

The height of each nickel pillar becomes equivalent to the thickness of the sacrificial layer. Each nickel pillar is shaped to have H = 15 μm and B” = 14 μm; the ratio of the height to the top diameter is near unity.

**Figure 7:**
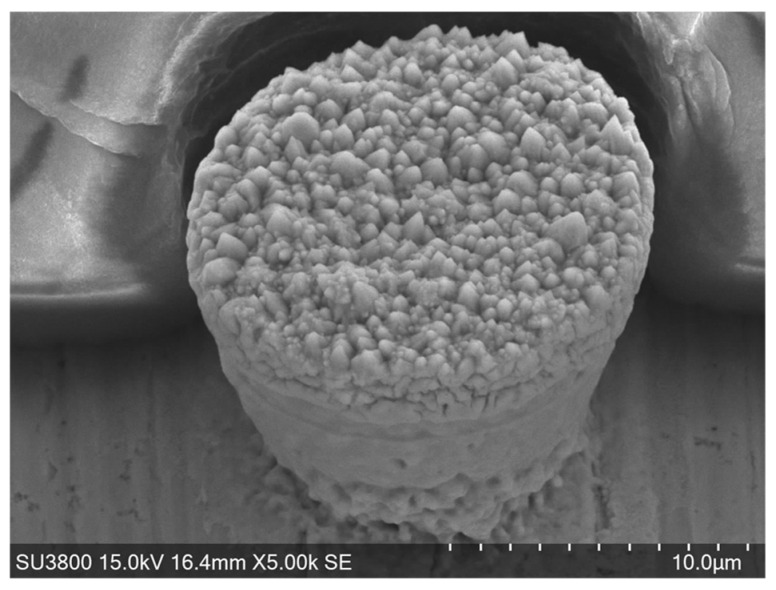
A micro-pillar unit cell after selectively nucleating and growing into the through-hole in the sacrificial film.

Figure 7 depicts a single micro-pillar unit cell. Since B” = B’ > B, this pillar has a paraboloidal surface corresponding to the inner surface of the through-hole in Figure 5. On the other hand, the bottom diameter of this micro-pillar is 8 μm, which is larger than B = 5 μm in Figure 5. The nickel cluster infiltrates into the interface between the sacrificial film and the copper sheet before growing to a certain height. This infiltration of nickel textures implies that the chemical solution soaks into the interface at the beginning of the wet-plating process.

**Figure 8:**
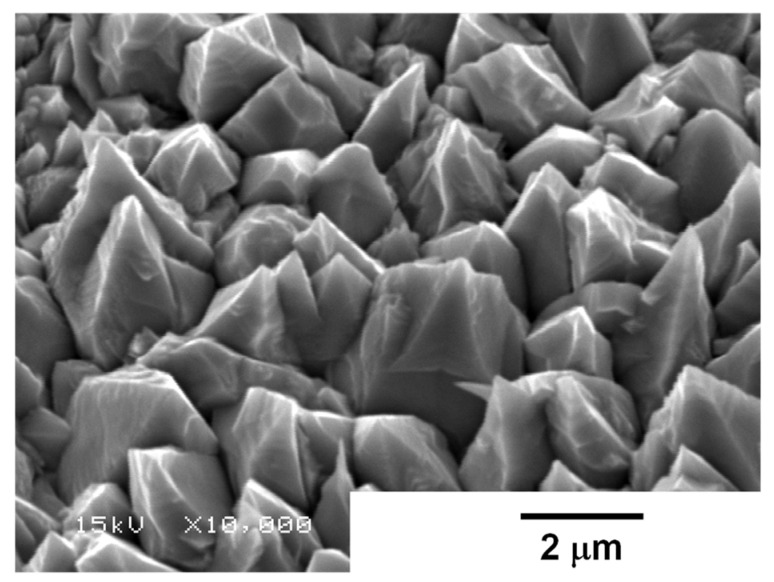
Dendritic growth of nickel deposits at the initial stage of the wet-plating process.

Let us investigate the growing process of nickel clusters inside of the through-hole. During the initial stage of wet plating, the process was stopped to describe the growth of nickel deposits on the copper sheet. As shown in Figure 8, every nickel deposit grew in a dendritic pattern without secondary dendritic growth on the copper sheet. The surface morphology at the top of the micro-pillar in Figure 7 simply corresponds to this dendritic growth. This shows that each nickel cluster grows in a dendritic pattern independently of one another and forms a sub-μm-sized pillar layer-by-layer. To be discussed later, this two-dimensional growth must be modified to control the unit cell geometry during the wet-plating process.

### 3.3. IR Emissivity of the Micro-Pillared Specimen

Thermographic microscopy with FT-IR was utilized to measure the IR emission spectrum of the micro-pillared copper specimen, as well as the bare copper sheet. This bare copper specimen was used as a reference, whereby its IR spectrum was treated as a baseline in the measurement of IR spectra for the microtextured copper specimen.

During measurement, the aperture in Figure 3 was set to 200 μm. The copper clearance between adjacent micro-pillars also makes a contribution to the measured IR spectrum. As discussed in [13], the intensity of IR spectra shows a trend of decreasing by itself due to the contribution of bare copper clearance with low intensities in the focused area to every IR spectrum measurement. Then, this IR spectrum for the bare copper sheet, or, I_Cu_ (λ), was first measured using the same aperture in the focused area on the copper specimen.

**Figure 9:**
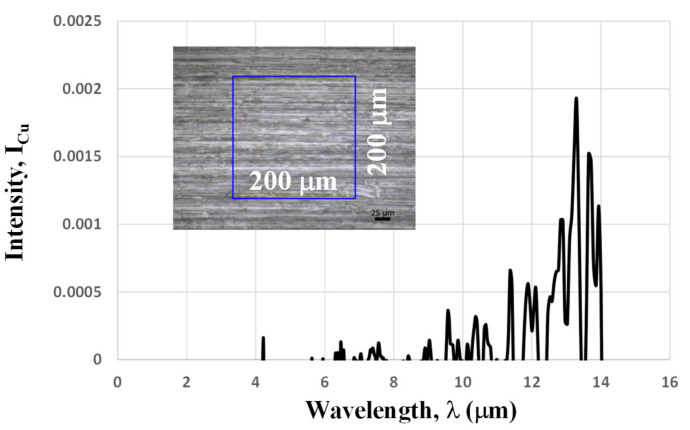
IR spectrum of the bare copper specimen to determine the baseline for the measurement of the IR emittance of the A-specimen.

**Figure 10:**
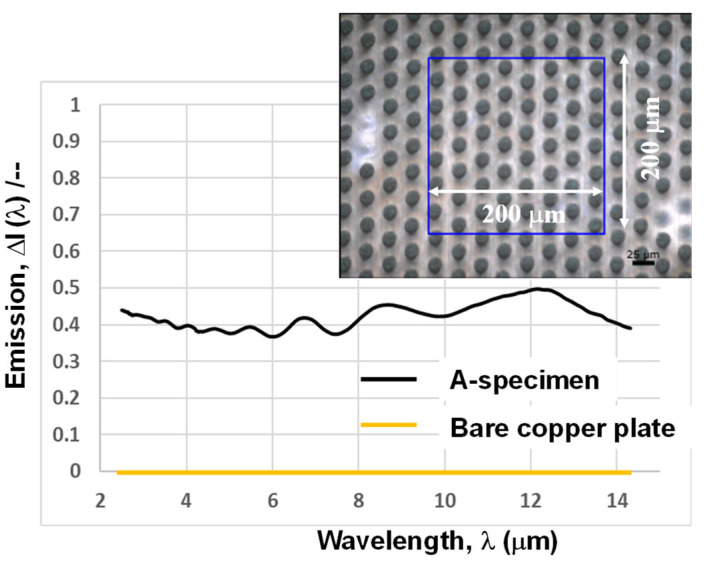
I_p_’ (λ) (= I_p_ (λ) − I_cu_ (λ)), contribution of the micro-pillar array to the IR emission.

As shown in Figure 9, this I_Cu_ (λ) has very low intensity, irrespective of the wavelength; e.g., the maximum intensity is less than 2 × 10^−3^. This low emissivity simply characterizes the IR optical performance of the polished copper sheet. In fact, as listed in [14], the emissivity at λ = 1.6 μm is 0.03, and it becomes much less for 8 μm < λ < 14 μm in the case of the polished copper sheet. In the following, this I_Cu_ (λ) is employed as a reference. The inserted image in Fig. 10 depicts the focused area for the measurement of the IR emission spectrum for the micro-pillared copper specimen. The aperture was fixed to focus the measurement area to 200 μm × 200 μm, in a similar manner to the measurement of I_Cu_ (λ) in Figure 9. Using I_Cu_ (λ) as a baseline, the measured IR emission spectrum I_p_ (λ) was processed by I_p_’ (λ) = I_p_ (λ) − I_Cu_ (λ) for 2 μm < λ < 14 μm.

Figure 10 shows the IR spectrum I_p_’ (λ) for the micro-pillared A-specimen with reference to the baseline. The intensity of I_p_’ (λ) becomes nearly constant by 0.4 to 0.5 irrespective of λ. Some resonance peaks are detected in I_p_’ (λ); each peak is so broad that the IR spectrum for the micro-pillared specimen becomes almost constant and insensitive to the wavelength. This broadening in the resonance peaks might come from the deviation in sub-pillar heights in a single unit cell, as partially discussed in [13].

As reported in [4,5], I (λ) for 2 < λ < 5 μm reached more than 0.90 when using the microtextured nano-photonic and meta-material devices. However, those IR spectra had significant wavelength dependency; e.g., I (λ) significantly decreased from 0.95 to 0.5 for λ > 5 μm. On the other hand, in the case of this micro-pillared specimen, I_p_’ (λ) is insensitive to the wavelength; e.g., I_p_’ (λ) only fluctuates from 0.45 to 0.5 even for λ > 10 μm.

In the literature, many studies have been reported to improve emission intensity through the modulation of unit cell structure and materials. As theoretically and experimentally studied in the following reference, the IR emissivity can be controlled by the phase change of the photonic structure [15]. When using the thin metallic glass film, the amorphous structure had an influence on the enhancement of IR emissivity, with it being compared to silicon and other metallic films [16]. This study proved that emission intensity reaches 0.45, nearly equal to the present data.

Let us consider this relatively low emissivity in I_p_’ (λ) for the micro-pillared specimen. As stated before, the copper clearances in the focused area make a negative contribution to reducing the emissivity. For simplicity, the measured emissivity is assumed to be proportional to the ratio (f) of the surface area of nickel microtextures to the focused area by aperture. In this triangular lattice structure in Figure 10, this ratio is calculated by the ratio of the top surface area (A_0_) of microtexture unit cells in Figure 7 to the regular triangle area (B_0_). The top view area of the micro-pillared unit cell is defined by (π/4) B”^2^. In the triangular lattice structure, half of this top view area contributes to this ratio, f. As stated in [17], the entire surface area of acicular microtextures becomes a double of this top view area; e.g., A_0_ = 2 × (π/4)B”^2^. On the other hand, B_0_ is defined by the regular triangle lattice area; e.g., B_0_ = D”^2^/{2 × (3)^1/2^}. Then,

f = A_0_/B_0_ = [2 × (π/4)B”^2^]/[D”^2^/{2 × (3)^1/2^}] (1)

Since B” = 14 μm and D” = 45 μm, f = 0.52. If the copper clearance area is reduced to zero, or, f → 1.0, the upper bound intensity of emissivity in average, I_p_ (1.0), is estimated by I_p_ (1.0) = (1.0/0.52) × I_p_ (0.52). The average of emissivity intensity for f = 0.52 is given by I_p_ (0.52) = 0.45 in Figure 10. Then, I_p_ (1.0) is estimated by 0.87. That is, without the copper clearances in the focused area, higher emissivity can be attained even by the nickel micro-pillared textures.

## 4. Discussion

Many methods have been developed to form micro- and nano-textured unit cell alignment onto polymer and metallic sheets. Precise mechanical machining with the use of a diamond chip [18] is adaptive to accurately control each unit cell size and geometry. A huge amount of machining time is needed to align the unit cell array into the large-area sheet. The laser interference technique provides the fastest and cheapest way to achieve the accurate alignment of unit cells irrespective of the sheet area [19]. The unit cell geometry is limited by the optical interactions among the beams. Femtosecond laser microtexturing is useful to control the unit cell size and topology, as well as its alignment, even in a large area sheet. The lithography also works as a method to align the micro-/nano-textured through-holes onto any metallic sheet [13,20]. The unit cell size can be minimized to μm or sub-μm orders, but its geometry is limited to being cylindrical or columnar. Table 1 compares the merits and demerits among the four approaches.

Each micro-pillar unit cell shape is determined by the energy profile of the femtosecond laser in machining, since each laser-machined through-hole shape simply corresponds to the Gaussian energy profile of the laser shot. Hence, by broadening this laser spot by defocusing in the laser micro-machining, each unit cell has a wider paraboloidal side surface to reduce the copper clearance. Then, higher emissivity is attained by f → 1.0 or by microtexturing the entire focused area in the IR emission measurement. In addition to this area ratio control, the morphology of microtextures also has an influence on the IR emissivity.

Various microtextures have been developed to control the emission of light and thermal radiation. Nano-precision microtextured CNTs and elastomers worked as a perfect blackbody with nearly zero emission [21]. Those microtextures have no resonance to light and IR waves in the specified wavelength range. Each microtexture, either convex or concave, is expected to control the directivity of IR wave emission [22]. The acicular pillars, or, the micro-honeycombs with parabolic reflective curved surfaces, can pixelate the directional IR emission. These studies suggest that IR emissivity can be varied by controlling the size, geometry, and morphology of the microtexturing unit cell and alignment. In the present study, the nickel micro-pillar with its aspect ratio of nearly 1.0 and paraboloidal side surface was fabricated as a unit cell via the femtosecond laser machining and wet-plating processes. These micro-pillars were aligned in the triangular lattice structure to increase the IR emissivity. The concave clearance among three paraboloidal unit cells in the lattice structure improves the emissivity, as suggested in [23,24].

Using femtosecond laser micromachining, a complex-shaped unit cell was designed and fabricated to control the IR emissivity even in the specified wavelength range. It is represented by an assembly of paraboloidal micro-pillars via nickel cluster deposition into the laser-machined through-hole unit cell pattern. As pointed out in [3,4,17], the wavelength range in the IR emission can be selected by the topological design of the unit cell. In those approaches, a single microtexture was utilized as a unit cell. In the present approach, the alignment is also controllable to modulate the unit cell morphology as well as the size of unit cells to improve the IR emissivity in the wider wavelength range.

Various polymer and inorganic films are available as work materials. The film thickness is also controllable only by coating the thicker film. This flexibility is preferable to the self-organization process via wet plating in comparison with other conventional self-organization processes [25].

The present combination of femtosecond laser machining with wet plating has several superiorities in the manufacturing of IR emission devices. Different from metal materials [6] and silicon-based systems [26], the IR emission device is made from metals and alloys; e.g., the present device consists of a copper sheet and pure nickel microtextures. This has sufficient engineering endurance to work even in the air. There is no limitation on its size and dimensions in principle. As reported in [17], A4-sized specimens can be obtained similarly, as stated above. This device can be equipped onto the parts, components, and systems to be cooled down through heat radiation. This flexible accommodation of the heat radiation device is useful for practical applications in industry and home facilities. With the present approach, laser micromachining to build up the through-hole array is firmly combined with wet plating to feed the nickel clusters into the array. It is possible to devise this firm combination of two processes into three steps. During the first step, the sacrificial film with the tailored through-hole array is fabricated using femtosecond laser micromachining. In the next step, this film is pasted onto the surface of metallic parts and components. Using this pasted film as a replica mold, the metallic microtextures are built up onto the objectives, filling the replica mold cavity with nickel clusters via wet plating during the final step.

## 5. Conclusions

A new manufacturing process is proposed to fabricate a micro-pillared IR radiation device with regular alignment on a copper sheet. Polyethylene film is coated onto the copper sheet with a specified thickness of 15 μm. This film is utilized as a sacrifice for femtosecond laser machining to cut the through-hole array with the regular triangle lattice structure. The through-hole is shaped to have a paraboloidal side surface and a micro-dimple on the interface between the sacrificial film and the copper sheet. This replica die is wet-plated only to feed the nickel clusters into the through-hole and to fully fill it. The micro-dimple works as a nucleation and growth site. After chemically removing the PE film and cleansing, the nickel micro-pillared unit cell with the paraboloidal surface is formed with a regular triangular lattice on the copper sheet.

This nickel micro-pillared sheet has high IR emissivity in a wide wavelength range; e.g., I (λ) = 0.4 to 0.5 for 2 μm < λ < 14 μm. By decreasing the contribution of copper clearance to emissivity, this I (λ) might well increase up to nearly 0.9. This demonstrates that a metallic microtextured device works as an IR resonator for efficient heat radiation in practice.

## Figures and Tables

**Table 1:** Comparison of merits and demerits among mechanical machining, the laser interference technique, lithography, and femtosecond laser microtexturing.

Procedure	Alignment	Unit Cell Size	Unit Cell Geometry	Duration	Cost
Mechanical Machining	Limited to small areas	Accurate as designed	Accurate as designed	Fast	Cheap
Laser Interference Technique	Applicable to large areas	Nearly Accurate	Not accurate	Fastest	Cheapest
Lithography	Applicable to large areas	Accurate	Limited by cylinder or column	Multi-step	Expensive
Femtosecond Laser Microtexturing	Applicable to large areas	Accurate As designed	Controllable	Faster	Normal

## Data Availability

Data are contained within the article.
